# 3-(3-Meth­oxy­phen­yl)benzo[*d*]thia­zolo[3,2-*a*]imidazol-9-ium hydrogen sulfate

**DOI:** 10.1107/S1600536812030541

**Published:** 2012-07-10

**Authors:** Hoong-Kun Fun, Tze Shyang Chia, Ahmed M. Alafeefy, Hatem A. Abdel-Aziz

**Affiliations:** a X-ray Crystallography Unit, School of Physics, Universiti Sains Malaysia, 11800 USM, Penang, Malaysia; b Department of Pharmaceutical Chemistry, College of Pharmacy, Salman Bin Abdulaziz University, PO Box 173, Alkharj 11942, Saudi Arabia; c Department of Pharmaceutical Chemistry, College of Pharmacy, King Saud University, PO Box 2457, Riyadh 11451, Saudi Arabia

## Abstract

In the title mol­ecular salt, C_16_H_13_N_2_OS^+^·HSO_4_^−^, the thia­zolo[3,2-*a*]benzimidazolium ring system is roughly planar [maximum deviation = 0.046 (3) Å] and makes a dihedral angle of 58.22 (11)° with the benzene ring. The meth­oxy group is almost coplanar with its attached benzene ring [C_meth­yl_—O—C—C = −1.6 (5)°]. In the crystal, the cation is linked to the anion by a bifurcated N—H⋯(O,O) hydrogen bond, generating an *R*_1_^2^(4) ring motif. The ion pairs are then connected by a C—H⋯O hydrogen bond into inversion dimers and these dimers are further linked by O—H⋯O hydrogen bonds into an infinite tape along [100]. A π–π stacking inter­action [centroid-to-centroid distance = 3.5738 (18) Å] and a short inter­molecular contact [S⋯O = 2.830 (3) Å] are also observed.

## Related literature

For the biological activities of thia­zolo[3,2-*a*]benzimidazoles, see: Chimirri *et al.* (1988 ▶); Al-Rashood & Abdel-Aziz (2010 ▶); Hamdy *et al.* (2007 ▶); Abdel-Aziz *et al.* (2007 ▶, 2008 ▶); Farag *et al.* (2011 ▶). For hydrogen-bond motifs, see: Bernstein *et al.* (1995 ▶). For the stability of the temperature controller used for the data collection, see: Cosier & Glazer (1986 ▶).
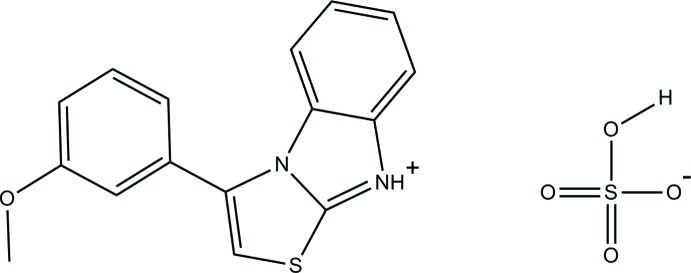


## Experimental

### 

#### Crystal data

C_16_H_13_N_2_OS^+^·HSO_4_^−^
*M* *_r_* = 378.41
Monoclinic, 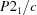

*a* = 4.5428 (8) Å
*b* = 20.096 (4) Å
*c* = 17.788 (3) Å
β = 93.003 (4)°
*V* = 1621.6 (5) Å^3^
*Z* = 4
Mo *K*α radiation
μ = 0.36 mm^−1^
*T* = 100 K
0.31 × 0.15 × 0.12 mm

#### Data collection

Bruker APEX DUO CCD diffractometer
Absorption correction: multi-scan (*SADABS*; Bruker, 2009 ▶) *T*_min_ = 0.898, *T*_max_ = 0.959
12491 measured reflections
3699 independent reflections
2904 reflections with *I* > 2σ(*I*)
*R*_int_ = 0.050

#### Refinement

*R*[*F* ^2^ > 2σ(*F* ^2^)] = 0.058
*wR*(*F* ^2^) = 0.170
*S* = 1.04
3699 reflections
231 parameters
H atoms treated by a mixture of independent and constrained refinement
Δρ_max_ = 0.99 e Å^−3^
Δρ_min_ = −0.68 e Å^−3^

### 

Data collection: *APEX2* (Bruker, 2009 ▶); cell refinement: *SAINT* (Bruker, 2009 ▶); data reduction: *SAINT*; program(s) used to solve structure: *SHELXTL* (Sheldrick, 2008 ▶); program(s) used to refine structure: *SHELXTL*; molecular graphics: *SHELXTL*; software used to prepare material for publication: *SHELXTL* and *PLATON* (Spek, 2009 ▶).

## Supplementary Material

Crystal structure: contains datablock(s) global, I. DOI: 10.1107/S1600536812030541/hb6885sup1.cif

Structure factors: contains datablock(s) I. DOI: 10.1107/S1600536812030541/hb6885Isup2.hkl

Supplementary material file. DOI: 10.1107/S1600536812030541/hb6885Isup3.cml

Additional supplementary materials:  crystallographic information; 3D view; checkCIF report

## Figures and Tables

**Table 1:** Hydrogen-bond geometry (Å, °)

*D*—H⋯*A*	*D*—H	H⋯*A*	*D*⋯*A*	*D*—H⋯*A*
N1—H1N1⋯O3	0.94 (4)	1.85 (4)	2.750 (3)	160 (3)
N1—H1N1⋯O4	0.94 (4)	2.50 (4)	3.199 (4)	132 (3)
O2—H1O2⋯O3^i^	0.97	1.60	2.531 (4)	158
C11—H11*A*⋯O5^ii^	0.93	2.32	3.237 (4)	170

Symmetry codes: (i) 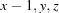; (ii) 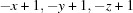.

**Fig. 1.**
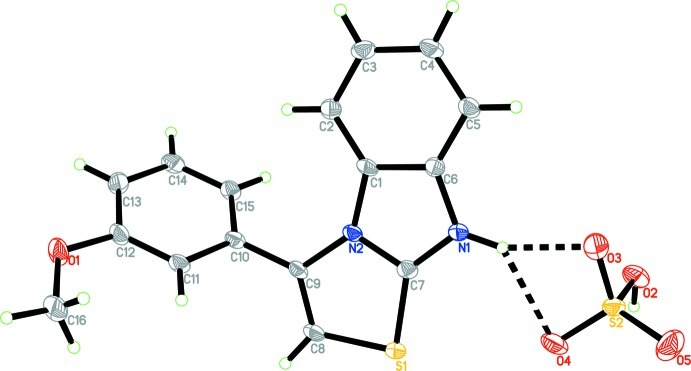
The molecular structure of the title compound with 50% probability displacement ellipsoids. Hydrogen bonds are indicated by dashed lines.

**Fig. 2.**
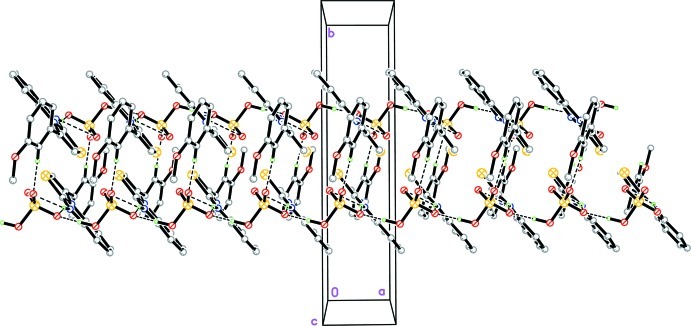
The crystal packing of the title compound. The dashed lines represent the hydrogen bonds. For clarity sake, hydrogen atoms not involved in hydrogen bonding have been omitted.

## supplementary crystallographic information

### Comment

Various biological properties of thiazolo[3,2-*a*]benzimidazole derivatives have been reported, including antibacterial, antifungal, anti-inflammatory, antiulcer, antiviral, anthelmintic and anticancer activities (Chimirri *et al.*, 1988; Al-Rashood & Abdel-Aziz, 2010). In continuation of our interest in this area (Hamdy *et al.*, 2007; Abdel-Aziz *et al.*, 2007, 2008; Farag *et al.*, 2011), we report herein the crystal structure of the title compound.

The molecular structure of the title compound is shown in Fig. 1. The asymmetric unit of the title compound, C_16_H_13_N_2_OS^+^, HSO_4_^-^, contains a 3-(3-methoxyphenyl)benzo[*d*]thiazolo[3,2-*a*]imidazol-9-ium cation and a hydrogen sulfate anion. The thiazolo[3,2-*a*]benzimidazole ring system is roughly planar [S1/N1/N2/C1–C9; maximum deviation = 0.046 (3) Å at atom C8] and makes a dihedral angle of 58.22 (11)° with the C10–C15 benzene ring. The O1—C16 methoxy group is almost coplanar with the C10–C15 benzene ring as indicated by the C16—O1—C12—C11 torsion angle of -1.6 (5)°.

In the crystal (Fig. 2), the cation and anion are linked to each other by bifurcated N1—H1N1···(O3,O4) hydrogen bonds, generating an *R*_1_^2^(4) ring motif (Bernstein *et al.*, 1995). The asymmetric units are then connected by C11—H11A···O5 hydrogen bond into inversion dimers and the dimers are further linked by O2—H1O2···O3 hydrogen bond into an infinite tape, propagating along the *a*-axis. A π–π interaction [*Cg*1···*Cg*2 = 3.5738 (18) Å; *Cg*1 and *Cg*2 are the centroids of N1/C6/C1/N2/C7 and C1–C6 rings, respectively; symmetry code = -1 + *x*, *y*, *z*] and a short intermolecular contact [S1···O4 = 2.830 (3) Å; symmetry code = -*x*, 1 - *y*, 1 - *z*] are also observed.

### Experimental

A mixture of 2-mercaptobenzimidazole (1.52 g, 10 mmol) and 3-methoxyacetophenone (1.52 g, 10 mmol) in boiling AcOH/H_2_SO_4_ (50 ml; 10:1, *v*/*v*) was refluxed for 5 h. The reaction mixture was then left to cool at room temperature. The colourless blocks formed were filtered off, washed with ethanol and dried to afford the title compound.

### Refinement

Atoms H1O2 and H1N1 were located in a difference Fourier map and the atom H1O2 was then fixed at its found location [O2—H1O2 = 0.9734 Å] and refined using a riding model with *U*_iso_(H) = 1.5*U*_eq_(O), whereas the atom H1N1 was refined freely [N1—H1N1 = 0.93 (4) Å]. The remaining H atoms were positioned geometrically [C—H = 0.93 and 0.96 Å] and refined with *U*_iso_(H) = 1.2 or 1.5*U*_eq_(C). A rotating group model was applied to the methyl group.

### Crystal data

**Table:**

C_16_H_13_N_2_OS^+^·HSO_4_^−^	*F*(000) = 784
*M**_r_* = 378.41	*D*_x_ = 1.550 Mg m^−^^3^
Monoclinic, *P*2_1_/*c*	Mo *K*α radiation, λ = 0.71073 Å
Hall symbol: -P 2ybc	Cell parameters from 4717 reflections
*a* = 4.5428 (8) Å	θ = 2.3–29.3°
*b* = 20.096 (4) Å	µ = 0.36 mm^−^^1^
*c* = 17.788 (3) Å	*T* = 100 K
β = 93.003 (4)°	Block, colourless
*V* = 1621.6 (5) Å^3^	0.31 × 0.15 × 0.12 mm
*Z* = 4	

### Data collection

**Table:**

Bruker APEX DUO CCD diffractometer	3699 independent reflections
Radiation source: fine-focus sealed tube	2904 reflections with *I* > 2σ(*I*)
Graphite monochromator	*R*_int_ = 0.050
φ and ω scans	θ_max_ = 27.5°, θ_min_ = 1.5°
Absorption correction: multi-scan (*SADABS*; Bruker, 2009)	*h* = −5→5
*T*_min_ = 0.898, *T*_max_ = 0.959	*k* = −26→24
12491 measured reflections	*l* = −19→23

### Refinement

**Table:**

Refinement on *F*^2^	Primary atom site location: structure-invariant direct methods
Least-squares matrix: full	Secondary atom site location: difference Fourier map
*R*[*F*^2^ > 2σ(*F*^2^)] = 0.058	Hydrogen site location: inferred from neighbouring sites
*wR*(*F*^2^) = 0.170	H atoms treated by a mixture of independent and constrained refinement
*S* = 1.04	*w* = 1/[σ^2^(*F*_o_^2^) + (0.0882*P*)^2^ + 3.0462*P*] where *P* = (*F*_o_^2^ + 2*F*_c_^2^)/3
3699 reflections	(Δ/σ)_max_ = 0.001
231 parameters	Δρ_max_ = 0.99 e Å^−^^3^
0 restraints	Δρ_min_ = −0.68 e Å^−^^3^

### Special details

**Table:**

Experimental. The crystal was placed in the cold stream of an Oxford Cryosystems Cobra open-flow nitrogen cryostat (Cosier & Glazer, 1986) operating at 100.0 (1) K.
Geometry. All e.s.d.'s (except the e.s.d. in the dihedral angle between two l.s. planes) are estimated using the full covariance matrix. The cell e.s.d.'s are taken into account individually in the estimation of e.s.d.'s in distances, angles and torsion angles; correlations between e.s.d.'s in cell parameters are only used when they are defined by crystal symmetry. An approximate (isotropic) treatment of cell e.s.d.'s is used for estimating e.s.d.'s involving l.s. planes.
Refinement. Refinement of *F*^2^ against ALL reflections. The weighted *R*-factor *wR* and goodness of fit *S* are based on *F*^2^, conventional *R*-factors *R* are based on *F*, with *F* set to zero for negative *F*^2^. The threshold expression of *F*^2^ > σ(*F*^2^) is used only for calculating *R*-factors(gt) *etc*. and is not relevant to the choice of reflections for refinement. *R*-factors based on *F*^2^ are statistically about twice as large as those based on *F*, and *R*-factors based on ALL data will be even larger.

### Fractional atomic coordinates and isotropic or equivalent isotropic displacement parameters (Å^2^)

**Table:**

	*x*	*y*	*z*	*U*_iso_*/*U*_eq_	
S1	0.21267 (17)	0.46487 (4)	0.42583 (4)	0.0192 (2)	
S2	0.24639 (17)	0.37855 (4)	0.66173 (4)	0.0214 (2)	
O1	0.7757 (6)	0.47885 (11)	0.06026 (13)	0.0313 (6)	
O2	0.0440 (5)	0.31645 (11)	0.67548 (13)	0.0281 (5)	
H1O2	−0.1640	0.3283	0.6741	0.042*	
O3	0.5152 (6)	0.34615 (13)	0.63785 (13)	0.0325 (6)	
O4	0.1087 (6)	0.41582 (12)	0.59898 (14)	0.0315 (6)	
O5	0.2805 (7)	0.41374 (13)	0.73091 (15)	0.0416 (7)	
N1	0.5806 (6)	0.36239 (12)	0.48611 (14)	0.0176 (5)	
N2	0.5187 (5)	0.37532 (11)	0.36286 (13)	0.0154 (5)	
C1	0.7199 (6)	0.32219 (14)	0.37436 (16)	0.0162 (6)	
C2	0.8648 (7)	0.28056 (14)	0.32574 (16)	0.0185 (6)	
H2A	0.8406	0.2849	0.2737	0.022*	
C3	1.0472 (7)	0.23229 (15)	0.35886 (17)	0.0217 (6)	
H3A	1.1477	0.2035	0.3282	0.026*	
C4	1.0841 (7)	0.22567 (15)	0.43704 (17)	0.0209 (6)	
H4A	1.2093	0.1927	0.4569	0.025*	
C5	0.9397 (7)	0.26676 (14)	0.48591 (17)	0.0195 (6)	
H5A	0.9654	0.2624	0.5379	0.023*	
C6	0.7538 (6)	0.31496 (14)	0.45269 (16)	0.0173 (6)	
C7	0.4426 (6)	0.39749 (14)	0.43096 (16)	0.0168 (6)	
C8	0.2254 (7)	0.46239 (14)	0.32772 (16)	0.0198 (6)	
H8A	0.1249	0.4926	0.2961	0.024*	
C9	0.3934 (6)	0.41295 (14)	0.30187 (15)	0.0167 (6)	
C10	0.4589 (6)	0.39786 (14)	0.22327 (16)	0.0171 (6)	
C11	0.5788 (7)	0.44837 (14)	0.18026 (16)	0.0186 (6)	
H11A	0.6104	0.4906	0.2006	0.022*	
C12	0.6502 (7)	0.43475 (15)	0.10674 (16)	0.0212 (6)	
C13	0.6007 (7)	0.37120 (15)	0.07654 (17)	0.0226 (6)	
H13A	0.6500	0.3621	0.0275	0.027*	
C14	0.4790 (7)	0.32211 (15)	0.11920 (17)	0.0212 (6)	
H14A	0.4460	0.2801	0.0986	0.025*	
C15	0.4049 (6)	0.33474 (14)	0.19277 (16)	0.0188 (6)	
H15A	0.3207	0.3016	0.2212	0.023*	
C16	0.8306 (10)	0.54428 (17)	0.0891 (2)	0.0367 (9)	
H16A	0.9177	0.5709	0.0513	0.055*	
H16B	0.9628	0.5418	0.1329	0.055*	
H16C	0.6481	0.5641	0.1023	0.055*	
H1N1	0.540 (8)	0.3670 (18)	0.537 (2)	0.022 (9)*	

### Atomic displacement parameters (Å^2^)

**Table:**

	*U*^11^	*U*^22^	*U*^33^	*U*^12^	*U*^13^	*U*^23^
S1	0.0257 (4)	0.0124 (4)	0.0195 (4)	0.0041 (3)	0.0020 (3)	−0.0003 (3)
S2	0.0273 (4)	0.0140 (4)	0.0233 (4)	0.0020 (3)	0.0053 (3)	−0.0002 (3)
O1	0.0577 (17)	0.0144 (11)	0.0228 (11)	−0.0097 (10)	0.0120 (11)	0.0003 (9)
O2	0.0312 (13)	0.0176 (11)	0.0352 (13)	−0.0015 (9)	−0.0016 (10)	0.0050 (9)
O3	0.0345 (14)	0.0369 (15)	0.0262 (12)	0.0089 (10)	0.0037 (10)	0.0035 (10)
O4	0.0381 (14)	0.0203 (12)	0.0365 (13)	0.0082 (10)	0.0046 (10)	0.0096 (10)
O5	0.067 (2)	0.0288 (14)	0.0294 (13)	−0.0083 (12)	0.0063 (12)	−0.0110 (11)
N1	0.0243 (13)	0.0119 (12)	0.0165 (12)	0.0029 (9)	0.0011 (9)	−0.0007 (9)
N2	0.0198 (12)	0.0094 (11)	0.0169 (11)	0.0001 (8)	−0.0001 (9)	0.0004 (9)
C1	0.0178 (14)	0.0089 (13)	0.0219 (14)	−0.0009 (10)	0.0012 (10)	0.0031 (10)
C2	0.0228 (15)	0.0137 (14)	0.0190 (13)	−0.0015 (11)	0.0013 (11)	−0.0008 (11)
C3	0.0260 (16)	0.0132 (14)	0.0262 (15)	0.0016 (11)	0.0047 (12)	−0.0031 (12)
C4	0.0249 (16)	0.0125 (14)	0.0252 (15)	0.0027 (11)	−0.0014 (11)	0.0020 (11)
C5	0.0242 (16)	0.0138 (14)	0.0203 (14)	0.0003 (11)	0.0001 (11)	0.0020 (11)
C6	0.0224 (15)	0.0123 (13)	0.0172 (13)	−0.0009 (10)	0.0024 (10)	0.0005 (11)
C7	0.0190 (14)	0.0119 (13)	0.0196 (14)	−0.0017 (10)	0.0011 (10)	−0.0004 (10)
C8	0.0269 (16)	0.0105 (14)	0.0218 (14)	0.0022 (11)	0.0005 (11)	0.0022 (11)
C9	0.0217 (15)	0.0102 (13)	0.0179 (14)	−0.0007 (10)	−0.0015 (10)	0.0029 (10)
C10	0.0204 (15)	0.0114 (13)	0.0192 (13)	0.0005 (10)	−0.0011 (10)	0.0012 (11)
C11	0.0254 (16)	0.0093 (13)	0.0209 (14)	−0.0018 (10)	−0.0005 (11)	0.0000 (11)
C12	0.0312 (17)	0.0132 (14)	0.0194 (14)	−0.0026 (11)	0.0024 (11)	0.0028 (11)
C13	0.0334 (18)	0.0165 (15)	0.0181 (14)	−0.0027 (12)	0.0027 (12)	−0.0036 (11)
C14	0.0261 (16)	0.0116 (14)	0.0256 (15)	−0.0029 (11)	−0.0021 (12)	−0.0018 (11)
C15	0.0236 (15)	0.0114 (13)	0.0214 (14)	−0.0038 (10)	0.0001 (11)	0.0000 (11)
C16	0.066 (3)	0.0121 (16)	0.0334 (19)	−0.0074 (15)	0.0170 (17)	0.0012 (13)

### Geometric parameters (Å, º)

**Table:**

S1—C7	1.710 (3)	C4—C5	1.388 (4)
S1—C8	1.750 (3)	C4—H4A	0.9300
S2—O5	1.421 (3)	C5—C6	1.396 (4)
S2—O4	1.458 (2)	C5—H5A	0.9300
S2—O3	1.466 (3)	C8—C9	1.348 (4)
S2—O2	1.577 (2)	C8—H8A	0.9300
O1—C12	1.358 (4)	C9—C10	1.476 (4)
O1—C16	1.428 (4)	C10—C15	1.396 (4)
O2—H1O2	0.9734	C10—C11	1.399 (4)
N1—C7	1.337 (4)	C11—C12	1.391 (4)
N1—C6	1.389 (4)	C11—H11A	0.9300
N1—H1N1	0.93 (4)	C12—C13	1.399 (4)
N2—C7	1.353 (4)	C13—C14	1.378 (4)
N2—C1	1.413 (4)	C13—H13A	0.9300
N2—C9	1.417 (3)	C14—C15	1.392 (4)
C1—C2	1.393 (4)	C14—H14A	0.9300
C1—C6	1.401 (4)	C15—H15A	0.9300
C2—C3	1.387 (4)	C16—H16A	0.9600
C2—H2A	0.9300	C16—H16B	0.9600
C3—C4	1.398 (4)	C16—H16C	0.9600
C3—H3A	0.9300		
			
C7—S1—C8	88.78 (14)	N1—C7—N2	110.6 (2)
O5—S2—O4	115.49 (16)	N1—C7—S1	135.9 (2)
O5—S2—O3	114.67 (17)	N2—C7—S1	113.4 (2)
O4—S2—O3	109.68 (14)	C9—C8—S1	114.2 (2)
O5—S2—O2	107.32 (15)	C9—C8—H8A	122.9
O4—S2—O2	107.17 (14)	S1—C8—H8A	122.9
O3—S2—O2	101.22 (15)	C8—C9—N2	110.1 (2)
C12—O1—C16	117.0 (2)	C8—C9—C10	128.3 (3)
S2—O2—H1O2	112.1	N2—C9—C10	121.5 (2)
C7—N1—C6	107.6 (2)	C15—C10—C11	120.8 (3)
C7—N1—H1N1	123 (2)	C15—C10—C9	121.0 (3)
C6—N1—H1N1	129 (2)	C11—C10—C9	118.2 (3)
C7—N2—C1	108.2 (2)	C12—C11—C10	119.2 (3)
C7—N2—C9	113.5 (2)	C12—C11—H11A	120.4
C1—N2—C9	138.1 (2)	C10—C11—H11A	120.4
C2—C1—C6	121.6 (3)	O1—C12—C11	124.8 (3)
C2—C1—N2	133.4 (3)	O1—C12—C13	115.2 (3)
C6—C1—N2	105.0 (2)	C11—C12—C13	120.0 (3)
C3—C2—C1	116.6 (3)	C14—C13—C12	120.2 (3)
C3—C2—H2A	121.7	C14—C13—H13A	119.9
C1—C2—H2A	121.7	C12—C13—H13A	119.9
C2—C3—C4	121.8 (3)	C13—C14—C15	120.7 (3)
C2—C3—H3A	119.1	C13—C14—H14A	119.6
C4—C3—H3A	119.1	C15—C14—H14A	119.6
C5—C4—C3	122.0 (3)	C14—C15—C10	119.0 (3)
C5—C4—H4A	119.0	C14—C15—H15A	120.5
C3—C4—H4A	119.0	C10—C15—H15A	120.5
C4—C5—C6	116.3 (3)	O1—C16—H16A	109.5
C4—C5—H5A	121.9	O1—C16—H16B	109.5
C6—C5—H5A	121.9	H16A—C16—H16B	109.5
N1—C6—C5	129.7 (3)	O1—C16—H16C	109.5
N1—C6—C1	108.6 (2)	H16A—C16—H16C	109.5
C5—C6—C1	121.7 (3)	H16B—C16—H16C	109.5
			
C7—N2—C1—C2	178.7 (3)	C8—S1—C7—N2	−0.4 (2)
C9—N2—C1—C2	−6.0 (6)	C7—S1—C8—C9	0.3 (2)
C7—N2—C1—C6	0.2 (3)	S1—C8—C9—N2	−0.1 (3)
C9—N2—C1—C6	175.4 (3)	S1—C8—C9—C10	−178.0 (2)
C6—C1—C2—C3	−0.8 (4)	C7—N2—C9—C8	−0.2 (3)
N2—C1—C2—C3	−179.2 (3)	C1—N2—C9—C8	−175.2 (3)
C1—C2—C3—C4	0.0 (4)	C7—N2—C9—C10	177.9 (3)
C2—C3—C4—C5	0.3 (5)	C1—N2—C9—C10	2.8 (5)
C3—C4—C5—C6	0.3 (4)	C8—C9—C10—C15	−125.6 (3)
C7—N1—C6—C5	179.7 (3)	N2—C9—C10—C15	56.7 (4)
C7—N1—C6—C1	0.4 (3)	C8—C9—C10—C11	54.9 (4)
C4—C5—C6—N1	179.6 (3)	N2—C9—C10—C11	−122.8 (3)
C4—C5—C6—C1	−1.2 (4)	C15—C10—C11—C12	−1.3 (4)
C2—C1—C6—N1	−179.1 (3)	C9—C10—C11—C12	178.2 (3)
N2—C1—C6—N1	−0.4 (3)	C16—O1—C12—C11	−1.6 (5)
C2—C1—C6—C5	1.5 (4)	C16—O1—C12—C13	−179.9 (3)
N2—C1—C6—C5	−179.8 (3)	C10—C11—C12—O1	−177.9 (3)
C6—N1—C7—N2	−0.3 (3)	C10—C11—C12—C13	0.3 (5)
C6—N1—C7—S1	−176.2 (3)	O1—C12—C13—C14	178.9 (3)
C1—N2—C7—N1	0.0 (3)	C11—C12—C13—C14	0.5 (5)
C9—N2—C7—N1	−176.5 (2)	C12—C13—C14—C15	−0.3 (5)
C1—N2—C7—S1	176.94 (19)	C13—C14—C15—C10	−0.8 (5)
C9—N2—C7—S1	0.4 (3)	C11—C10—C15—C14	1.6 (4)
C8—S1—C7—N1	175.4 (3)	C9—C10—C15—C14	−177.9 (3)

### Hydrogen-bond geometry (Å, º)

**Table:**

*D*—H···*A*	*D*—H	H···*A*	*D*···*A*	*D*—H···*A*
N1—H1*N*1···O3	0.94 (4)	1.85 (4)	2.750 (3)	160 (3)
N1—H1*N*1···O4	0.94 (4)	2.50 (4)	3.199 (4)	132 (3)
O2—H1*O*2···O3^i^	0.97	1.60	2.531 (4)	158
C11—H11*A*···O5^ii^	0.93	2.32	3.237 (4)	170

Symmetry codes: (i) *x*−1, *y*, *z*; (ii) −*x*+1, −*y*+1, −*z*+1.
